# The Labor Question

**Published:** 1888-05

**Authors:** 


### HALL'S JOURNAL OF HEALTH

TRUTH DEMANDS NO SACRIFICE; ERROR CAN MAKE NONE.

VOL. 35. MAY, 1888. No. 5.

### THE LABOR QUESTION.

In spite of the fine spun theories of our political economists, things go on between labor and capital, pretty much in the old way. Capital always has been as it is likely always to be, master of the situation. The various attempts of labor to gain the upper hand, or at least, to put itself upon an equality with its co-operator, have invariably failed, because when it comes to an open rupture, the issue is narrowed to a question of endurance, in which capital holds the vantage ground. It represents the well fortified position, amply armed and provisioned for a long siege, whilst the mobolized and unprovided beseigers, can at best, only hold out for a limited time ere they are forced to give up the contest from very exhaustion, and things necessarily fall into the old ways.

But there is something radically wrong in the workings of our social system, when men and women of ability holding subordinate positions in the various avenues and industries open to them, are forced to labor all the available hours of the day, and oftentimes, far into the night in positions requiring the exercise of integrity, skill and exactitude, for grossly inadequate compensation, whilst the profits of their aggregate labor are enriching a few capitalists who are thus enabled to live a life of idleness and disipation.

He who is born rich and uses his capital selfishly, is as much of a lord in this country as in any other. Many a costly equipage rolls through the avenues of our Central Park on a pleasant afternoon, carrying a parvenu, whose wealth has been mainly accumulated from the products of sewing girls, whose weekly pay for from twelve to fifteen hours daily of life consuming labor, averages not more than three dollars.

Is it surprising that the laboring classes are constantly endeavoring to release themselves from this species of bondage? It is to be regretted that all efforts to bring about a state of affairs, more consistent with right and justice, have hitherto met with failure.

There are nevertheless some noble exceptions to the common rule, on the part of employers. It is becoming quite common to concede to workman a share of the net profits accumulated by their industry, and we know of no such arrangement, which has not been attended with good results. Under such a rule, every one feels that he has an interest in the success of the enterprise in which his labor and skill are enlisted, beyond his stated wages, for upon its economical management his extra earnings depend. Experience has demonstrated that it is entirely possible to conduct extensive operations upon this plan. It is one that is founded in justice, and should be adopted wherever practicable, that capital and labor, always necessarily united, may be more closely and fraternally allied. They should stand side by side, lock-handed. All the projects which advance our civilization, should no less ameliorate the condition of the struggling masses. Indeed one follows the other of a necessity, since that which conserves not the public good, is a positive injury to the state.

Co-operative enterprises have not succeeded so well. One cause of failure has been a lack of capital. Another is a lack of head. Where all have an equal voice there is likely to be much jealousy and fault finding with the selected managers, sometimes glaringly incompetent, who are held blamable for every mishap.

On something of this plan, with a fixed financial head, the experiment is being tried on "Topolobampo Bay," Gulf of California, Mexico, of building up a city in the wilderness, with no sustaining surroundings, if we omit what is claimed to be an excellent harbor with abundance of fish and water fowl. We shall watch this project with a good deal of interest, and trust that our sister Republic, with which our country has not always been on the best of terms, will treat it tenderly, especially in respect to conscriptions wherewith to carry on its military operations. One of its recent advocates claims for it, superior advantages on account of its isolation. This feature does not usually, conduce to the growth of cities, nor so far as we know, have towns located in the wilderness prospered to any great extent. It is too, a novel feature that this company, with all the elements of a municipality, is incorporated under the laws of the State of Colorado, and established with its proprietory interests in Sinaloa, Mexico. It does not appear that any special privileges have been secured to it from the Mexican Government, or any inhibition of its methods of adding to its own revenues. To a lawyer this state of affairs would seem to be frought with some danger. But the enthusiast is not likely to let such trifles stand in his way. Hitherto it has not been much of a rage for Americans to become Mexican Citizens and even Mr. Owen will pardon us for expressing some doubts of its advantages. They ought surely to be more substantial than cheap land, a mild climate and a wide reach of sea coast, under a system which precludes private ownership, and the transmission of estates. We would not advise any one to exchange a present comfortable home and citizenship for an interest in this occidental Utopia. At best it is an experiment, which the poor cannot afford, and the rich do not want.

